# Estimating Forest Aboveground Biomass by Combining Optical and SAR Data: A Case Study in Genhe, Inner Mongolia, China

**DOI:** 10.3390/s16060834

**Published:** 2016-06-07

**Authors:** Zhenfeng Shao, Linjing Zhang

**Affiliations:** State Key Laboratory of Information Engineering in Surveying, Mapping and Remote Sensing, Wuhan University, 129 Luoyu Road, Wuhan 430079, China; shaozhenfeng@whu.edu.cn

**Keywords:** biomass estimation, Landsat 8 OLI, RADARSAT-2, combined vegetation index, prediction method, sample size

## Abstract

Estimation of forest aboveground biomass is critical for regional carbon policies and sustainable forest management. Passive optical remote sensing and active microwave remote sensing both play an important role in the monitoring of forest biomass. However, optical spectral reflectance is saturated in relatively dense vegetation areas, and microwave backscattering is significantly influenced by the underlying soil when the vegetation coverage is low. Both of these conditions decrease the estimation accuracy of forest biomass. A new optical and microwave integrated vegetation index (VI) was proposed based on observations from both field experiments and satellite (Landsat 8 Operational Land Imager (OLI) and RADARSAT-2) data. According to the difference in interaction between the multispectral reflectance and microwave backscattering signatures with biomass, the combined VI (COVI) was designed using the weighted optical optimized soil-adjusted vegetation index (OSAVI) and microwave horizontally transmitted and vertically received signal (HV) to overcome the disadvantages of both data types. The performance of the COVI was evaluated by comparison with those of the sole optical data, Synthetic Aperture Radar (SAR) data, and the simple combination of independent optical and SAR variables. The most accurate performance was obtained by the models based on the COVI and optical and microwave optimal variables excluding OSAVI and HV, in combination with a random forest algorithm and the largest number of reference samples. The results also revealed that the predictive accuracy depended highly on the statistical method and the number of sample units. The validation indicated that this integrated method of determining the new VI is a good synergistic way to combine both optical and microwave information for the accurate estimation of forest biomass.

## 1. Introduction

Forest aboveground biomass (AGB) accounts for the dominant share of terrestrial biomass stocks [1]. There is a strong need for estimating forest AGB across large spatial scales. For example, estimates of forest AGB support sustainable forest management, bioenergy production and the detection of land-use change. Furthermore, the assessment of carbon stocks for global climate change modeling and initiatives such as Reducing Emissions from Deforestation and Forest Degradation (REDD) and REDD+ rely on forest AGB estimates [2,3]. During the last decades valuable remote sensing tools have been developed to measure AGB in time and space.

Various types of remotely sensed data have been used to estimate AGB [4,5,6,7,8]. Multispectral images are among the most spatiotemporally available, since multispectral spaceborne sensors are the most widely used and longest-operating sensor types, which is important for regional AGB monitoring [9]. Multispectral optical sensors can easily distinguish the basic distribution of green vegetation, which concerns the photosynthesis of plants. Moreover, the multispectral vegetation indices (VIs) have been widely used for fine vegetation classification and the estimate of biophysical and biochemical parameters. However, a high estimation accuracy is difficult to achieve due to spectral saturation problems in dense forest [10,11], and passive optical remote sensing signal can be largely obscured by cloud cover and significant atmospheric aerosol interference.

Over the past two decades, a large number of studies have shown that forest biomass can be retrieved using SAR data [12,13,14]. SAR data is independent from clouds and atmospheric aerosols, is sensitive to plant structure, and depending on the used frequency, is capable of penetrating vegetation [15,16,17]. Many radar-based biomass estimation studies used L-band SAR data, especially the Advanced Land Observing Satellite (ALOS) Phased Array type L-band Synthetic Aperture Radar (PALSAR) L-band data [18,19,20]. This is because long wavelength L-band SAR data has a stronger capability to penetrate forest canopy and capture more information about the vertical structure of the forest canopy. However, the ALOS satellite stopped operating in April 2011 and cannot provide L-band SAR data after 2011. The successful launch of RADARSAT-2 in 2007, which is equipped with a fully polarized SAR operating at C-Band (5.3 GHz) with a wavelength of approximately 5.6 cm, has provided a new opportunity for the use of radar to estimate AGB. Some studies [21,22] showed significant correlations between C-band backscatter and forest structural parameters such as diameter-at-breast height (DBH), volume, basal area, height, and AGB. However, estimating AGB using the relationship between biomass and SAR backscattering remains problematic due to not only high sensitivity to soil conditions, including surface roughness and soil moisture in low vegetation coverage, but also to saturation at high biomass levels [14,23].

Light detection and ranging (LiDAR) data can provide detailed vegetation structure measurements at discrete locations covering circular or elliptical footprints from a few centimeters to tens of meters in diameter [24]. LiDAR can obtain accurate AGB estimations for various forests with no saturation at higher biomass levels [25,26]. However, LiDAR systems are often limited to airborne acquisition, which is better suited to providing samples (e.g., transects) rather than full wall-to-wall coverage over large areas [27,28]. Even though LiDAR provides the best estimates of forest biomass, observations in overlarge areas remain problematic and expensive. Therefore, our study focused on the use of optical and radar data.

Given the advantages and weaknesses of optical and radar data and the need to overcome the limitations associated with the use of either data type alone, we expect that a combination of optical and SAR data is of high value for aboveground biomass modeling. In fact, some promising results have also been obtained using combined optical and SAR information for the estimation of biomass or other vegetation parameters. Attarchi *et al.* [29] combined Landsat ETM+ bands, ALOS PALSAR backscattering and their derived features using multiple linear regression models to improve the estimation accuracy of forest AGB. Häme *et al.* [30] used a combination of ALOS PALSAR backscattering and ALOS AVNIR spectral data to improve the performance of biomass estimation based on multiple linear regression models. Most previous studies used a simple combination of optical and SAR data [29,30,31], that is to say, independent optical and SAR variables were simultaneously considered as predictor variables. However, the specific mathematical and physical significance of this method for combining optical and SAR data has not been considered.

AGB has been remotely estimated using a variety of approaches, including parametric (e.g., regression models) and nonparametric approaches (e.g., decision-/regression-tree models) [6,32,33,34,35,36]. In recent years, more flexible, often nonparametric methods from the fields of geostatistics and machine learning have become more prevalent. Methods such as K-nearest Neighbor (KNN), Support Vector Machine (SVM), Back Propagation Neural Networks (BPNN) and Random Forest (RF) methods have a higher potential than standard regression models due to the ability to identify complex nonlinear relationships between response and predictor variables [34,37,38]. Furthermore, the lack of prior knowledge about correlation structures for complex data [20,39] leads to an increased application of nonparametric methods. This shortage of knowledge is particularly evident when combining various remote sensing predictors from different sensors (*i.e.*, ranging from spectral indices to predictors related to SAR backscatter intensity) [34].

In this study, we tested the synergic use of optical (Landsat 8 OLI) and radar (RADARSAT-2) remote sensing data to improve the estimation accuracy of forest biomass. First, five widely-used statistical prediction methods were applied to optical data and microwave data separately, as well as to the simple combination of independent optical and microwave data, across a range of sample sizes. In order to examine the effect of predictor variable selection on biomass estimation accuracy, the optimal variables for optical and microwave data were identified by a stepwise selection procedure and then applied to the same prediction algorithms and sample sizes used in first step. Finally, a combined VI (COVI) incorporating both optical and microwave information was proposed and the model performance based on the new COVI was evaluated by comparison with those based on all other tested variables. Our aim is to present an enhanced synergic inversion method that accurately determines forest aboveground biomass from remote sensing data.

## 2. Materials and Methods

### 2.1. Study Area

**Figure 1:**
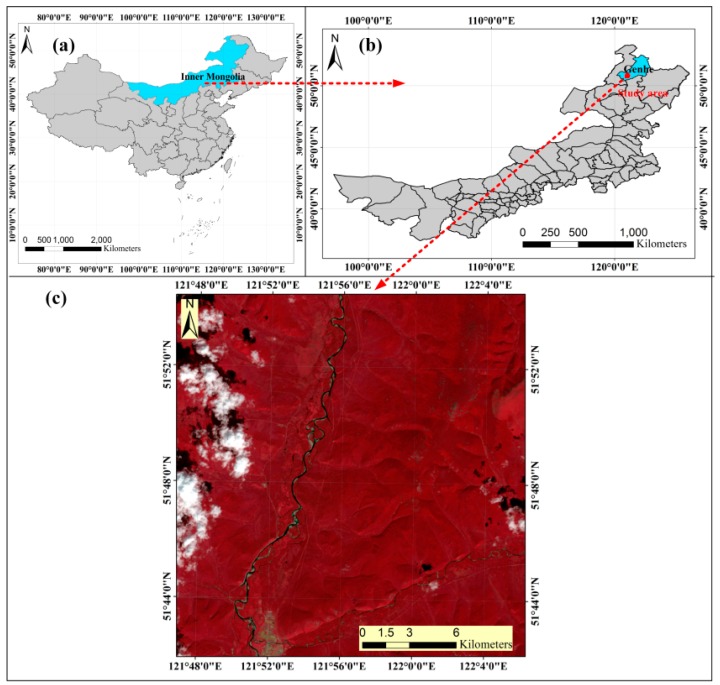
The study area. (**a**) The location of Inner Mongolia Autonomous Region in China; (**b**) The administrative boundary of Genhe in Inner Mongolia Autonomous Region, and the location of the study area in Genhe; (**c**) Landsat 8 OLI image (band 5, 4, 3 false color combination;8% cloud cover) acquired on 25August 2013 of the study area.

The study site focused on the Genhe area (50°48′N, 121°34′E, Figure 1) in the northeast of the Inner Mongolia Autonomous Region, China. It covers an area of approximately 625 km^2^ (25 km × 25 km). Cold temperate climatic conditions prevail, with a rainy season occurring from June to August with a mean annual rainfall of 464 mm. The mean annual temperature is approximately−5.3 °C. The forests of this study site mainly consist of native coniferous and broad-leaved species spreading along a topographically gentle terrain dominated by *Betula* and *Larix* spp.

### 2.2. Field Measurements and Aboveground Biomass Computation

The field data collection was conducted from 25 June to 29 August 2013. We measured tree DBH and tree height (H) and identified all tree species on 181 plots. DBH and H were used to generate *in-situ* AGB estimates to train the remote AGB-retrieval models and assess estimation accuracy, and they were measured using the Haglof Digitech Calliper and Vertex IV laser instrument, respectively. In this study, trees with DBH ≥3 cm were measured. The measurements were done using a grid-based systematic sampling technique, with approx. 30 m × 30 m sample plot, distributed systematically within the stand. Based on the DBH and H, the aboveground biomass was calculated for each composition (stock, branch and leaf) of the individual tree by applying species-specific allometric equations developed for the main forest in the northeast of China [40]. The biomass value for a single tree was estimated by summing up the biomass values of each tree component. The biomass values of the individual trees were finally summed up to obtain plot-specific biomass values in megagrams per hectare. The biomass values varied between 14.31 Mg/ha and 278.09 Mg/ha with a mean value of 112.59 Mg/ha and a standard deviation of 71.62 Mg/ha.

### 2.3. Remote Sensing Data Acquisition and Pre-Processing

The study utilized both Landsat 8 OLI and RADARSAT-2 satellite images. Landsat images are among the most frequently used medium spatial-resolution data for remote forest AGB estimation at local and regional scales [9,41]. RADARSAT-2, carrying a fully polarized SAR operating at C-Band (5.3 GHz) with a wavelength of approximately 5.6 cm, has opened up new opportunities for remote forest AGB estimation by the use of backscattering coefficients at different polarizations [21,22]. Landsat 8 OLI and RADARSAT-2 satellite images that covered the region of interest were acquired during the time that most closely coincided with the field measurement dates (*i.e.*, the 25 August 2013 and the 30 June 2013, respectively). The Landsat 8 OLI image was obtained with 8% cloud cover, sun azimuth angle of 155.84 and sun elevation angle of 47.07. It has a resolution of 30 m × 30 m for the non-panchromatic OLI bands. RADARSAT-2 data, which was acquired under conditions of a sunny and clear sky with less cloud cover than Landsat 8 OLI, was in a single look complex (SLC) format with a mean incident angle of 36.6. The fine quad-polarization wide (FQW) mode with 8 m nominal spatial resolution was selected for this study.

The Landsat 8 OLI image was obtained in digital number (DN) and needed to be converted to reflectance values. Preprocessing was performed in ENVI 5.1 software. Firstly, conversion from DN to Top-Of-Atmosphere (TOA) spectral radiances was implemented, following the approach described on the USGS website. Then the atmospheric correction of Landsat 8 OLI image to surface reflectance was performed using the Fast Line-of-sight Atmospheric Analysis of Spectral Hypercube (FLAASH). Prior to the analysis, the image was finally scrutinized for accurate geo-referencing using field-based GPS reference points (e.g., road intersections, bridges, rock formations). Further correction of Landsat 8 OLI image was not necessary.

Preprocessing of RADARSAT-2 data was achieved using the Next European Space Agency (ESA) SAR Toolbox (NEST) software. RADARSAT-2 image was first radiometrically calibrated to obtain the linear radar backscattering coefficients transformed from the DN. A 3 × 3 boxcar filter was then applied to the backscattering coefficients data to reduce speckle noise. Boxcar averaging adds N adjacent samples, divides the sum by N, and then writes that value into the Nth sample location and is suitable in homogeneous areas such as large forest areas. Subsequently, the image was geometrically corrected using the pre-processed Landsat 8 OLI image with an overall error of approximately 0.65 pixels. After that, RADARSAT-2 data was resampled to the same spatial resolution of Landsat 8 OLI image (30 m). Landsat 8 OLI and RADARSAT-2 needed to cover the entire and accurate extent of the study area. The two images were thus resized, resulting in a complete and precise coverage of the test site (25 km × 25 km).

### 2.4. Regression Algorithms

Different statistical and machine-learning techniques have been applied to model complex relationships between remote sensing signals and forest biomass [32,33,34,35,36]. In our study, we compared five parametric or nonparametric prediction models in terms of their accuracy for biomass estimation: KNN, SVM, BPNN, RF and Stepwise Linear Regression (LMSTEP).

RF is an ensemble learning algorithm [42], designed to improve the classification and regression trees (CART) method by integrating a large set of decision trees based on a deterministic technique, by selecting a random set of variables and a random sample from the training dataset. RF technique has furthermore become popular in remote sensing as a nonlinear and non-parametric alternative, with promising predictive capabilities for high-dimensional datasets [43]. Fassnacht *et al.* [34] showed that the RF was a suitable modeling technique for the estimation of forest aboveground biomass using hyperspectral and LiDAR data.

Generally speaking, in a KNN method the weighted mean of the response value from the most similar neighbor(s) is assigned to a target unit of interest, where the similarity is defined in a feature space consisting of candidate predictor variables [44]. The KNN technique has received considerable attention due to its ease of availability, and some previous studies revealed that the KNN technique has the potential to increase the precision of vegetation parameters estimates [45,46,47]. The KNN was used and compared with other models by Tian *et al.* [37] with respect to their predictive power of forest aboveground biomass and a relatively good performance of KNN was found in comparison to other models.

Support vector regression (SVR) is a training approach based on the framework of statistical learning theory. The main idea behind SVR is to estimate the linear dependency between pairs of n-dimensional input vectors and 1-dimensional target variables by fitting an optimal approximating hyperplane to a set of training samples. The method has proven its robustness to dimensionality and generalization ability [48]. As applied to remote sensing data, SVR has shown great capability to predict biophysical/chemical plant parameters by relating spectral information to *in situ* samples [49,50,51].

BPNN is one of the most popular and proven neural networks [52,53]. This machine-learning algorithm has great potential for estimating vegetation parameters due to its ability to find and learn complex linear and nonlinear relationships between radiometric data and vegetation parameters [52,54]. Zheng *et al.* [53] found that BPNN was better at predicting forest growing stock volume compared to LMSTEP based on Landsat Thematic Mapper (TM) and field data.

LMSTEP is a classical parametric method. The method aims at automatically choosing a set of predictive explanatory variables among all possible variables, and the partial F-statistic is a common criterion, with α as a threshold for adding or removing a predictor variable. We therefore include this technique in order to assess whether more flexible and complex nonparametric methods could outweigh the more frequently-used LMSTEP technique, which accounts for linear relationships between response and predictor variables.

### 2.5. Experiments

Using all the algorithms, we conducted three experiments (Experiments 1–3) with different data type combinations (Table 1). Predictors used in these experiments varied, depending upon available sensor spectral (or backscatter coefficients) data. Experiments were as follows: Experiment 1 was composed of three subtests: (i) Landsat 8 OLI image bands and image-derived variables; (ii) RADARSAT-2 backscatter coefficients and image-derived variables; and (iii) the joined variables from subtests (i) and (ii); Experiment 2 that also encompassed three subtests: (i) optical optimal variables chosen by the predictor selection method; (ii) SAR optimal variables; and (iii) the joined variables from subtests (i) and (ii); and Experiment 3: the *COVI* and optical and SAR optimal variables. Methods for the predictor selection and the *COVI* construction are detailed in Section 2.6 and Section 2.7, respectively. The following is an overview about the modeling set-up in Experiments 1–3. Four main processing steps were included:

(I) After preparing an original dataset of response and predictor variables, the dataset of size n was ordered according to ascending biomass values and was then subdivided in five equal-size subsets of size ns = n/5.
(II) Using the stratified dataset derived from step I, we developed subsamples for each stratum using bootstrapping [55]. For each of the four desired sample sizes, 100 datasets were drawn. The bootstrapping procedure incorporates the effects of sampling variability. The stratification ensures that samples from the full range of available biomass values were included in each bootstrapped input dataset. To build the four desired sample size classes, the number of sample units x drawn from each of the five subsets was varied four times (x = ns/4, x = ns/3, x = ns/2, x = ns). Input datasets of Class 1 (x = ns/4) consisted of the fewest number of sample units, while those of Class 4 (x = ns) contained the most.
(III) For each sample size, the 100 input datasets were fit by five prediction approaches including KNN, SVM, BPNN, RF and LMSTEP. More details on the prediction methods are provided in Section 2.4.
(IV) For each combination of predictor variables, prediction method and sample size, a cross validation (number of folds = 5, number of repetitions = 5) was applied. The validation method resulted in (1) obtaining the best model parameters (indicated by the model with lowest RMSE) and (2) retrieving diagnostics of RMSE and R^2^.

In addition to the model diagnostics, predictive models were fitted to obtain spatial prediction maps of biomass for visual comparison. All statistical calculations were implemented using the R statistical package [56].

### 2.6. Predictor Selection

**Figure 2:**
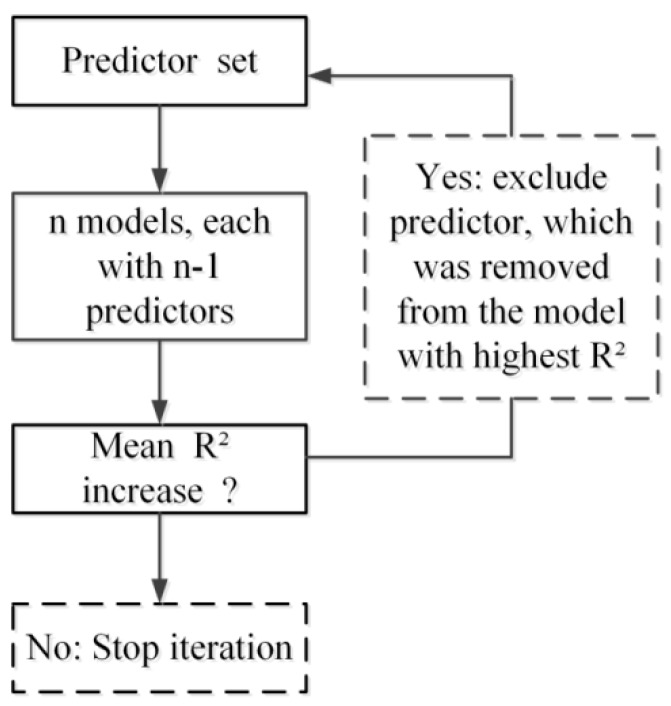
Flow chart of the stepwise predictor exclusion.

To examine the effect of predictor selection on model performances and identify more suitable predictors to obtain higher model accuracy, a stepwise procedure was used to exclude relatively poor predictors and highly intercorrelated predictors. We emulated the predictor selection method used in the study of Kattenborn *et al.* [35]. In each step, n models (n = number of predictors) were run using all but one predictor variable (n − 1).The model validation described above in Section 2.5 was applied to each model. Therefore, the model which had the best performance (R^2^) indirectly indicates the (excluded) predictor with the lowest explanatory power. In the next step, this predictor was removed from the set of candidate predictors. Model performance was evaluated using average values of the different model methods (KNN, SVM, BPNN, RF and LMSTEP). This procedure was continued until convergence of the R^2^ value was reached (Figure 2). The stepwise procedure was used to generate optical and SAR optimal predictor variables, respectively (*i.e.*, OOVs in Experiment 2 (i) and SOVs in Experiment 2 (ii)).

### 2.7. Combined Vegetation Index Construction

As stated before, both optical and SAR data have advantages and shortcomings in monitoring vegetation parameters. In order to overcome the limitations associated with the use of a single data type, a combined VI incorporating weighted optical and microwave information was designed and its equation can be written as:

(1) COVI=A×OVI+(1−A)×MBI

where COVI is the combined VI, OVI and MBI are the most important optical and microwave predictor variables chosen, respectively. A is the weighting factor and its calculation is elaborated below.

(1) After preparing a matrix of response and the most important optical (OVI) and microwave (MBI) predictor variables, the dataset of size n was first ordered according to ascending biomass values. OVI and MBI then needed to be normalized to allow for direct comparison between optical and microwave variables, e.g., with different scales and dynamic ranges.
(2) With limited and discrete samples, the sensitivity value of the predictor variable to the response variation could be expressed via first difference quotient method. This method consists of forward, backward and central difference quotients, as an approximation of the first derivative method [57,58]. In the study, the central difference quotient approach was applied to calculate the sensitivity value of OVI (or MBI) to biomass changes. Take OVI as an example, the processing can be carried out as:
  (2) $${\mathrm{OS}}_{i}=|\frac{\mathrm{OVI}\_n_{i+1}-\mathrm{OVI}\_n_{i-1}}{{\mathrm{AGB}}_{i+1}-{\mathrm{AGB}}_{i-1}}|\;(i=2,3,4...,n-1)$$
  where OVI_*n_i_* is the normalized value of OVI and OS*_i_* is the corresponding sensitivity value of case *i*.
(3) Based on the above analysis, the weights of optical data (*i.e.*, OVI) can be formulated as follows:
  (3) $$A_{i}={\mathrm{OS}}_{i}/({\mathrm{OS}}_{i}+{\mathrm{MS}}_{i})$$
  where *A_i_* is the weight value of OVI in case *i*, *MS_i_* is the sensitivity value of *MBI* under the *i’th* condition Equation (2).

In order to obtain the quantitative expression of the weight of optical (or microwave) data (*i.e.*, *A*), the relationship between the weight value of optical data and corresponding OVI value was then developed by statistical regression analysis.

## 3. Results

### 3.1. Experiments 1 and 2 Performances

**Figure 3:**
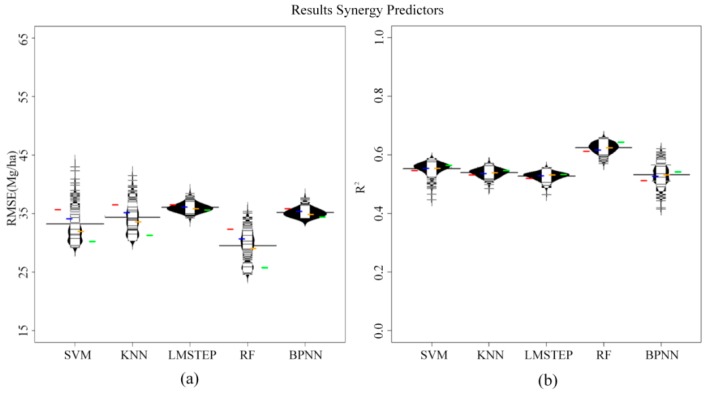
The beanplots illustrate the distribution of the mean RMSE (**a**); and R^2^ (**b**) values from the 100 bootstrapped models obtained by the 5-fold-cross validation for each prediction method and sample size with optical and microwave integrated dataset in Experiment 1 (iii). Furthermore, the median values of the corresponding accuracy measures for each of the four sample size classes are given with the colored horizontal stripes (Class 1 to Class 4 from left to right in colors red, blue, yellow and green).

**Figure 4:**
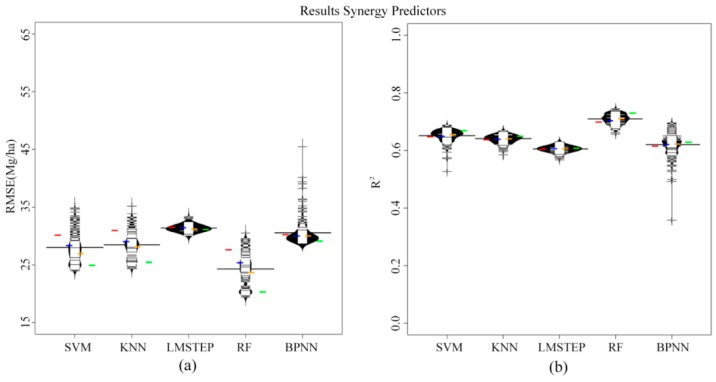
The beanplots illustrate the distribution of the mean RMSE (**a**); and R^2^ (**b**) values from the 100 bootstrapped models based on the combined optical and microwave optimal variables in Experiment 2 (iii). Explanations follow those from Figure 3.

The stepwise selection method retained seven predictors of optical sensor type, *i.e.*, SWIR bands; SR, NDVI, OSAVI, MSI and NDWI. The microwave optimal variables chosen by the selection procedure included: VV, HV, HH/HV and RVI. In Experiments 1 and 2 performed with sole optical or SAR metrics (*i.e.*, Experiments1 (i), (ii) and 2 (i), (ii)), the results were always not satisfactory with RMSE > 30 Mg/ha and R^2^ < 0.6, regardless of what prediction algorithm or sample size was used. Additionally, the employment of optical variables generally led to slightly smaller RMSE and higher R^2^ values compared to the microwave-only results. The differences in RMSE and R^2^ between the best optical result and the best SAR result were about 5 Mg/ha and 0.08, respectively. Figure 3 and Figure 4 summarize the model performances based on optical and microwave integrated dataset from Experiments 1 (iii) and 2 (iii), respectively. The use of the combined optical and microwave dataset resulted in better AGB estimates compared to the use of either the sole optical data or the sole microwave data, regardless of the prediction algorithm or sample size that was used. Furthermore, a relatively stable increase in R^2^ and decrease in RMSE were observed along with increasing sample size (colored horizontal lines from left to right). RF performed best compared to all other tested methods, especially when large numbers of sample units were available, and LMSTEP, in most cases, turned out slightly higher RMSE and lower R^2^ values when compared to all other methods.

From Figure 3 and Figure 4, we can also see that using the selected optimal variables resulted in relatively higher predictive performances of models (Experiment 2) compared to those using original tested predictors (Experiment 1). For instance, for RF, the differences in RMSE and R^2^ between the models based on combined optical and microwave optimal variables (*i.e.*, OOVs + SOVs in Experiment 2 (iii) in Figure 4) and those using a combination of original optical and microwave variables (*i.e.*, OVs + SVs in Experiment 1 (iii) in Figure 3) reached approximately 5 Mg/ha and 0.085.

### 3.2. Experiment 3 Performance

According to the results from previous tests, the simple combination of optical and microwave dataset (*i.e.*, OVs + SVs in Experiment 1 (iii) or OOVs + SOVs in Experiment 2 (iii)) improved results compared to either the optical-only or microwave-only data, regardless of what sample size or prediction algorithm was used, both before and after predictor variables selection. In order to further improve biomass estimation accuracy, a new *VI* incorporating optical and microwave information was developed. In subsequent analyses, we focused on the preferable RF-based methodology with optical and microwave optimal variables and the largest sample size.

From the previous experiments, the most important optical and microwave variables detected by RF were OSAVI and HV, which were therefore employed in the COVI computation. More details on the method of constructing the COVI were provided in Section 2.7. When setting up the new *VI*, we developed a subsample using stratified bootstrapping as well: the dataset of size n (181) ranked on the basis of rising biomass values was divided into ten equal-size subsets of size ns = n/10; one-sixth of each of the ten subsets was then extracted and was subsequently gathered up to obtain the subsample. Lastly, the 100 input datasets with the largest sample size (Class 4), whose predictor variables contained the COVI and residual optical and microwave optimal variables (*i.e.*, SWIR bands, SR, NDVI, MSI, NDWI and VV, HH/HV, RVI), were fit by RF prediction method.

#### 3.2.1. Combined Vegetation Index

**Figure 5:**
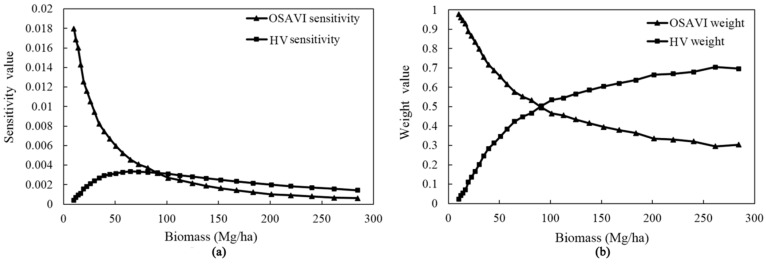
Sensitivity variation (**a**); and weight variation (**b**) of OSAVI and HV with increasing biomass.

Figure 5a shows the sensitivities of OSAVI and HV to biomass change. From this figure we can see that the HV sensitivity values rose in the first stage, reached a maximum of about 0.004, and then slowly declined with the increasing biomass. Meanwhile, as the biomass increased, there was a dramatic decrease in the sensitivity of OSAVI. Furthermore, the OSAVI was found to have a higher sensitivity to biomass variation than the microwave backscatter coefficient (HV), but the saturation problem of the optical data was also obvious. When biomass values were less than approximately 100 Mg/ha, OSAVI was more sensitive to the change of biomass than HV and played a more important role in the COVI. In contrast, the sensitivity values of HV outperformed those of OSAVI when biomass values exceeded about 100 Mg/ha, and thus the COVI mainly depended on HV information. Therefore, the scatterplot of OSAVI (or HV) weight values and biomass values (Figure 5b) indicates that the biomass value of about 100 Mg/ha could be perceived as a threshold: optical information largely controlled the *COVI* when biomass values were less than 100 Mg/ha and microwave information became increasingly important accompanied by increasing biomass values.

The quantitative regression relationship was finally developed using the weight of optical data (*i.e.*, A) as the dependent variable and corresponding OSAVI as the independent variable. Overall, the mathematical expression of the COVI can be shown as:

(4) $$\mathrm{COVI}=((-0.846)\times {\text{OVI}}^{2}-1.008\times \text{OVI}+1.293)\times \mathrm{OVI}+(1-((-0.846)\times {\text{OVI}}^{2}-1.008\times \text{OVI}+1.293))\times \mathrm{MBI}$$

where OVI and MBI are OSAVI and HV, respectively.

#### 3.2.2. Model Performances

According to the results in Experiments 1 and 2, the models (*i.e.*, NBM in Table 2) based on a simple combination of optical and microwave optimal variables (*i.e.*, OOVs + SOVs in Experiment 2(iii)) with RF method and the largest sample size (Class 4) performed best compared to all other tested combinations. Table 2 shows that the models in Experiment 3 (*i.e.*, BM) using the predictor set that included the COVI and residual optical and microwave optimal variables with the same prediction method (RF) and sample size (Class 4) generated better RMSE and R^2^ compared to NBM. The best-performing model reached a mean R^2^ of 0.82 with a mean RMSE of 15.95 Mg/ha and RMSE_r_ of 14.17%, and the latter achieved a mean R^2^ of 0.75 with a mean RMSE of 21.03 Mg/ha and RMSE_r_ of 18.68%.

### 3.3. Wall-to-Wall Predictions

**Figure 6:**
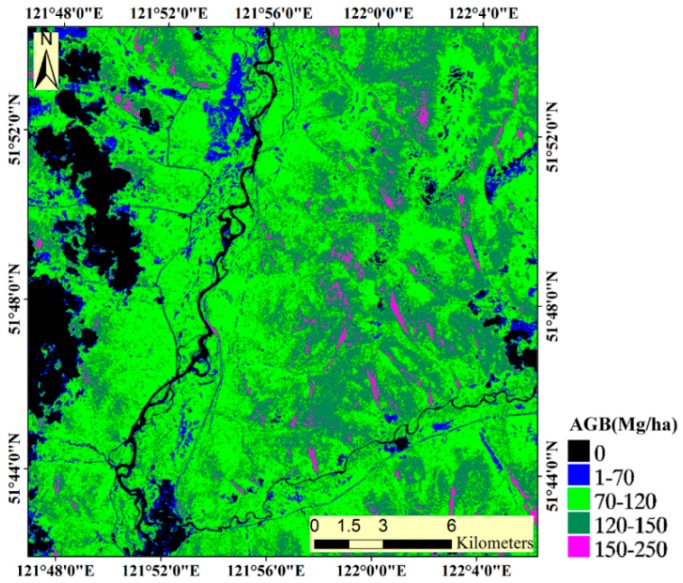
Wall-to-wall map of mean biomass estimates as obtained from the 100 bootstrapped model runs, using the *COVI* and residual optical and microwave optimal variables, random forest and largest sample size (models in Experiment 3).

Figure 6 illustrates the wall-to-wall mean biomass predictions as obtained from the best-performing models (*i.e.*, BM) based on the *COVI* and residual optical and microwave optimal variables using the largest number of sample units (Class 4) and RF method. The mean biomass estimates were reasonably well spread over the original range of reference biomass values. The predicted values varied from 14.68 Mg/ha to 249.67 Mg/ha, with a mean value of 106.45 Mg/ha. In most of the study area, the biomass values were in the range from 70 to 120 Mg/ha. Furthermore, many areas with low predicted AGB were situated near villages or major roads. The rivers and cloud cover areas have been masked out and other non-forested areas could be identified as homogeneous areas of low predicted biomass estimates.

## 4. Discussion and Conclusions

### 4.1. Performance of Sensor, Statistical Model and Sample Size

The results of Experiments 1 and 2 show that the sole use of either microwave data or optical data did not provide satisfactory results, and the simple combinations of independent optical and microwave data improved results compared to either the optical-only or microwave-only data, regardless of which prediction algorithm or sample size was used, before and after predictor variables selection. This is in line with earlier findings [15,59,60]. Attarchi *et al.* [29] evaluated 11 different multiple linear regression models using optical and L-band SAR variables, and demonstrated that only the joint use of SAR and multispectral data allowed a good estimation of AGB in those regions. The use of both optical and microwave information confirms our initial hypothesis that a combination of the two types of data enhances biomass estimations. This is because in the area with relatively dense vegetation coverage, the efficiency of multispectral data is affected by the saturation phenomenon. In contrast, C-band SAR data has penetration depth of few centimetres and is sensitive to top canopy architecture [21]. In addition, the sensitivity of C-band backscatter to forest AGB could also be partly attributed to heterogeneity in the forest canopy structures [21]. Cougo *et al.* [22] evaluated the relationships between backscattering of a RADARSAT-2 image and the structural attributes of regenerating mangrove vegetation, and found significant relationships between backscattering coefficients in VH, HH and VV and the average height, DBH and AGB which was mainly in the range from 40 to 200 Mg/ha. Chand *et al.* [21] also showed reasonable correlations between backscatter values derived from C-band SAR data and forest biometric parameters. In our study, according to the sensitivity analysis of optical VI (OSAVI) and SAR backscattering (HV) to biomass variation (Figure 5a), the sensitivity values of HV outperformed those of OSAVI when biomass values exceeded about 100 Mg/ha. Overall, we therefore conclude that C-band SAR signal, which is sensitive to canopy patterns, could provide important information that is not present in optical data, and their combination is valuable for accurate biomass estimation.

Although the objective of this study was not to compare algorithms, the study has shown that among the considered methods, RF outperformed the other tested approaches, particularly when many sample units were used. The good performance has also been found in other studies. Fassnacht *et al.* [34] showed that RF was better at predicting forest aboveground biomass compared to other tested methods based on hyperspectral and LiDAR data. The RF was also used and compared with other models by Latifi *et al.* [36] for the estimation of aboveground forest biomass and it proved to be superior to the other examined methods. We attribute the outstanding predictive accuracy to the flexibility and robustness of the RF approach, which markedly differs from all other tested methods due to its conceptual design. A problem with RF may be that the applied subsampling may lead to considerable variance of the estimates when applied to a small number of sample units (compare results regarding sample size). In most cases, LMSTEP performed worse than other tested algorithms, especially when applied to optical data. This phenomenon can be explained by the fact that relationships between optical predictors and observed biomass are likely nonlinear and thus not well fitted by LMSTEP.

Our results also indicate that the number of sample units is important for explaining the variance in R^2^ and RMSE on the test site. This is in accordance with other studies [34]. Figure 3 and Figure 4 show that a practically relevant effect on R^2^ and RMSE can be attributed to sample size (compare colored stripes), especially for RF.

### 4.2. Combined Vegetation Index

The best-performing model achieved in this study was based on the COVI and residual optical and microwave optimal variables. In fact, some promising results have been obtained by combining optical with SAR data to estimate biomass and other vegetation parameters [29,30,31]. Usually, a simple combination of the two types of data is used. For instance, the common method is that independent optical and SAR variables are simultaneously considered as predictor variables. Parametric or nonparametric relationships are developed between these variables and the predictive parameter, which is similar to the way of OVs + SVs in Experiment 1 (iii) (or OOVs + SOVs in Experiment 2 (iii)). However, the theoretically combined mechanism has not been considered in this case. In this study, a combined VI was proposed based on the optical reflectance and SAR backscatter mechanism, which determined the contributions of optical and microwave information under different biomass conditions. In other words, vegetation indices with fixed expressions are improved by the new VI with variable weights of optical and microwave data as biomass variation. From the comparison analysis of the 5-fold cross-validation (Table 2), it can be assumed that the models (*i.e.*, BM) based on the COVI and residual optical and microwave optimal variables outperformed the corresponding models (*i.e.*, NBM) using a simple combination of optical and microwave optimal variables (*i.e.*, OOVs + SOVs) and obtained the best predictive performance. The only difference between BM and NBM is the combined method of OSAVI and HV. OSAVI and HV was integrated through a simple combination in NBM, while in BM, OSAVI and HV was combined using the proposed synergic method resulting in the new *VI* (*i.e.*, COVI). We therefore conclude that the COVI can yield a significant improvement in the biomass estimation compared to a simple combination of independent SAR (*i.e.*, HV) and optical variables (*i.e.*, OSAVI). The high accuracy in estimating AGB can be attributed to the more specific mathematical and physical significance of the COVI due to the reasonable way of incorporating weighted optical and microwave information. Therefore, the COVI has valuable potential to enhance aboveground biomass estimation accuracy. However, further tests and validation are needed before the wide application of COVI.

### 4.3. Overall Performance of the Biomass Model

We concede that C-band SAR data has weaker penetrability than longer wavelength radar, such as L- and P-band data, which could lead to a negative effect on capturing vertical vegetation structure information, especially in dense vegetation coverage areas. We therefore suspect that one of the reasons why the models in the study have good performances was that the reference data in this study was concentrated on low-medium biomass values, with few field samples in high value ranges (more than 200 Mg/ha). The accuracy of the biomass estimation (R^2^ = 0.82, RMSE = 15.95 Mg/ha, RMSE_r_ = 14.17%) using the models based on the COVI and residual optical and microwave optimal variables (Experiment 3) in our study is comparable to or better than those of other similar studies. For example, Kattenborn *et al.* [35] evaluated the potential of data fusion based on hyperspectral and InSAR data using RF for temperate forest biomass estimation with R^2^ of 0.73 and RMSE_r_ of 15%. In the study of Fassnacht *et al.* [34], the data combination based on hyperspectral and airborne LiDAR data was performed to estimate forest biomass with a mean R^2^ of 0.72 and RMSE_r_ of 28.46%. Laurin [61] estimated tropical forest biomass using the integration of airborne LiDAR metrics with hyperspectral bands with R^2^ of 0.7 and RMSE_r_ of 35.83%.

In conclusion, the combined VI (COVI) provides a good synergistic way to integrate optical with microwave information. Therefore, the COVI presented could be useful in biomass and other vegetation parameters estimation. This study may be valuable in guiding further research on quantifying the different responses of optical reflectance and microwave backscatter to biomass and other vegetation variables. For the future, our synergistic method should be expanded to other forest types and sensor systems, especially the introduction of longer wavelength SAR data, for further validation and wide application.

## Figures and Tables

**Table 1:** Landsat 8 OLI and RADARSAT-2 predictor variables used in forest AGB estimation.

Data Type	Data Source	Details	Experiment	
Optical variables (OVs) ^a^	Landsat 8 OLI	blue, green, red, nearin, SWIRI, SWIRII, SR, NDVI, EVI, SAVI, SAVI2, MSAVI, OSAVI, MSI, Clgreen, NDWI1, NDWI2	(i)	1
SAR variables (SVs) ^b^	RADARSAT-2	VV, HH, VH, HV, VV/HH, HH/HV, VV/HV, RVI	(ii)	
OVs + SVs	Landsat 8 OLI, RADARSAT-2	(blue, green, red, nearin, SWIRI, SWIRII, SR, NDVI, EVI, SAVI1, SAVI2, MSAVI, OSAVI2, MSI, Clgreen, NDWI1, NDWI2) + (VV, HH, VH, HV, VV/HH, HH/HV, VV/HV, RVI)	(iii)	
optical optimal variables chosen (OOVs)	Landsat 8 OLI		(i)	2
SAR optimal variables chosen (SOVs)	RADARSAT-2		(ii)	
OOVs + SOVs	Landsat 8 OLI, RADARSAT-2		(iii)	
COVI + OOVs + SOVs	Landsat 8 OLI, RADARSAT-2		3	

^a^ SR: Simple Ratio Vegetation Index; NDVI: Normalized Difference Vegetation Index; EVI: Enhanced Vegetation Index; SAVI: Soil Adjusted Vegetation Index; SAVI2: Soil Adjusted Vegetation Index; MSAVI: Modified Soil Adjusted Vegetation Index; OSAVI: Optimized Soil-Adjusted Vegetation Index; MSI: Moisture Stress Index; Clgreen: Green chlorophyll index; NDWI1: Normalized Difference Water Index; NDWI2: Normalized Difference Water Index; ^b^ HH: the normalized radar cross-section (NRCS) measured from the horizontally transmitted and horizontally received signal; VV: the NRCS measured from the vertically transmitted and vertically received signal; HV and VH: the vertically transmitted and horizontally received signal; VV/HH, HH/HV, VV/HV: Polarization Ratio; RVI: Radar Vegetation Index, RVI = 8 * HV/(HH + VV + 2 * HV).

**Table 2:** Performances of the models (BM) based on *COVI* and residual optical and microwave optimal variables in Experiment 3 and the models (NBM) which performed best in Experiments 1 and 2.

Model Abbreviation	Predictors	Regression Algorithm	Sample Size	R^2^	RMSE (Mg/ha)	RMSE_r_ (%)
BM	COVI + residual optical and microwave optimal variables (*i.e.*, SWIR bands, SR, NDVI, MSI, NDWI and VV, HH/HV, RVI)	RF	Class 4	0.82	15.95	14.17
NBM	Simple combination of optical and microwave optimal variables (*i.e.*,OOVs + SOVs in Experiment 2 (iii))	RF	Class 4	0.75	21.03	18.68

## References

[1] Houghton R.A., Hall F., Goetz S.J. (2009). Importance of biomass in the global carbon cycle. J. Geophys. Res..

[2] Gibbs H.K., Brown S., Niles J.O., Foley J.A. (2007). Monitoring and estimating tropical forest carbon stocks: Making REDD a reality. Environ. Res. Lett..

[3] Koch B. (2010). Status and future of laser scanning, synthetic aperture radar and hyperspectral remote sensing data for forest biomass assessment. ISPRS J. Photogramm. Remote Sens..

[4] Tanase M.A., Panciera R., Lowell K., Tian S., Hacker J.M., Walker J.P. (2014). Airborne Multi Temporal L-band Polarimetric SAR Data for Biomass Estimation in Semi-Arid Forests. Remote Sens. Environ..

[5] Næsset E., Gobakken T., Bollandsås O.M., Gregoire T.G., Nelson R., Ståhl G. (2013). Comparison of precision of biomass estimates in regional field sample surveys and airborne LiDAR-assisted surveys in Hedmark County, Norway. Remote Sens. Environ..

[6] Morel A.C., Fisher J.B., Malhi Y. (2012). Evaluating the potential to monitor aboveground biomass in forest and oil palm in Sabah, Malaysia, for 2000–2008 with Landsat ETM+ and ALOS-PALSAR. Int. J. Remote Sens..

[7] Saatchi S., Marlier M., Chazdon R.L., Clark D.B., Russell A.E. (2011). Impact of spatial variability of tropical forest structure on radar estimation of aboveground biomass. Remote Sen. Environ..

[8] Li X., Yeh A.G.O., Wang S., Liu K., Liu X., Qian J., Chen X. (2007). Regression and analytical models for estimating mangrove wetland biomass in South China using Radarsat images. Int. J. Remote Sens..

[9] Gleason C.J., Im J.H. (2013). A Review of Remote Sensing of Forest Biomass and Biofuel: Options for Small-Area Applications. Gisci. Remote Sens..

[10] Lu D. (2005). Aboveground biomass estimation using Landsat TM data in the Brazilian Amazon. Int. J. Remote Sens..

[11] Mutanga O., Adam E., Cho M.A. (2012). High density biomass estimation for wetland vegetation using WorldView-2 imagery and random forest regression algorithm. Int. J. Appl. Earth Observ. Geoinf..

[12] Dobson M.C., Ulaby F.T., Letoan T., Beaudoin A., Kasischke E.S., Christensen N. (1992). Dependence of radar backscatter on conifer forest biomass. IEEE Trans. Geosci. Remote Sens..

[13] Sandberg G., Ulander L.M.H., Fransson J.E.S., Holmgren J., Toan T.L. (2011). L- and P-band backscatter intensity for biomass retrieval in hemiboreal forest. Remote Sens. Environ..

[14] Castel T., Beaudoin A., Stach N., Stussi N., Toan T.L., Durand P. (2001). Sensitivity of space-borne SAR data to forest parameters over sloping terrain. Theory and experiment. Int. J. Remote Sens..

[15] Gao S., Niu Z., Huang N., Hou X. (2013). Estimating the Leaf Area Index, height and biomass of maize using HJ-1 and RADARSAT-2. Int. J. Appl. Earth Observ. Geoinf..

[16] Ulaby F.T., Allen C.T., Eger G., Kanemasu E. (1984). Relating the microwave backscattering coefficient to leaf area index. Remote Sens. Environ..

[17] Inoue Y., Kurosu T., Maeno H., Uratsuka S., Kozu T., Dabrowska-Zielinska K., Qi J. (2002). Season-long daily measurements of multifrequency (Ka, Ku, X, C, and L) and full-polarization backscatter signatures over paddy rice field and their relationship with biological variables. Remote Sens. Environ..

[18] Mitchard E.T.A., Saatchi S.S., Lewis S.L., Feldpausch T.R., Woodhouse I.H., Sonké B., Rowland C., Meir P. (2011). Measuring biomass changes due to woody encroachment and deforestation/degradation in a forest–savanna boundary region of central Africa using multi-temporal L-band radar backscatter. Remote Sens. Environ..

[19] Rahman M.M., Sumantyo J.T.S. (2012). Retrieval of tropical forest biomass information from ALOS PALSAR data. Geocarto Int..

[20] Carreiras J.M.B., Vasconcelos M.J., Lucas R.M. (2012). Understanding the relationship between aboveground biomass and ALOS PALSAR data in the forests of Guinea-Bissau (West Africa). Remote Sens. Environ..

[21] Chand T.R.K., Badarinath K.V.S. (2007). Analysis of ENVISAT ASAR data for forest parameter retrieval and forest type classification—A case study over deciduous forests of central India. Int. J. Remote Sens..

[22] Cougo M., Souzafilho P., Silva A., Fernandes M., Santos J., Abreu M., Nascimento W., Simard M. (2015). Radarsat-2 Backscattering for the Modeling of Biophysical Parameters of Regenerating Mangrove Forests. Remote Sens..

[23] He Q., Cao C., Chen E., Sun G., Ling F., Pang Y., Zhang H., Ni W., Xu M., Li Z. (2012). Forest stand biomass estimation using ALOS PALSAR data based on LiDAR-derived prior knowledge in the Qilian Mountain, western China. Int. J. Remote Sens..

[24] Montesano P.M., Cook B.D., Sun G., Simard M., Nelson R.F., Ranson K.J., Zhang Z., Luthcke S. (2013). Achieving accuracy requirements for forest biomass mapping: A spaceborne data fusion method for estimating forest biomass and LiDAR sampling error. Remote Sens. Environ..

[25] Næsset E., Gobakken T. (2008). Estimation of above- and below-ground biomass across regions of the boreal forest zone using airborne laser. Remote Sens. Environ..

[26] Zhao K., Popescu S., Nelson R. (2009). Lidar remote sensing of forest biomass: A scale-invariant estimation approach using airborne lasers. Remote Sens. Environ..

[27] Mitchard E.T.A., Saatchi S.S., White L.J.T., Abernethy K.A., Jeffery K.J., Lewis S.L., Collins M., Lefsky M.A., Leal M.E., Woodhouse I.H. (2011). Mapping tropical forest biomass with radar and spaceborne LiDAR in Lopé National Park, Gabon: Overcoming problems of high biomass and persistent cloud. Biogeosciences.

[28] Boudreau J., Nelson R.F., Margolis H.A., Beaudoin A., Guindon L., Kimes D.S. (2008). Regional aboveground forest biomass using airborne and spaceborne LiDAR in Québec. Remote Sens. Environ..

[29] Attarchi S., Gloaguen R. (2014). Improving the Estimation of Above Ground Biomass Using Dual Polarimetric PALSAR and ETM+ Data in the Hyrcanian Mountain Forest (Iran). Remote Sens..

[30] Hame T., Rauste Y., Antropov O., Ahola H.A., Kilpi J. (2013). Improved Mapping of Tropical Forests With Optical and SAR Imagery, Part II: Above Ground Biomass Estimation. IEEE J. Sel. Top. Appl. Earth Observ. Remote Sens..

[31] Laurin G.V., Liesenberg V., Chen Q., Guerriero L., Frate F.D., Bartolini A., Coomes D., Wilebore B., Lindsell J., Valentini R. (2013). Optical and SAR sensor synergies for forest and land cover mapping in a tropical site in West Africa. Int. J. Appl. Earth Observ. Geoinf..

[32] Güneralp İ., Filippi A.M., Randall J. (2014). Estimation of floodplain aboveground biomass using multispectral remote sensing and nonparametric modeling. Int. J. Appl. Earth Observ. Geoinf..

[33] Dube T., Mutanga O., Dube T., Mutanga O. (2015). Evaluating the utility of the medium-spatial resolution Landsat 8 multispectral sensor in quantifying aboveground biomass in uMgeni catchment, South Africa. ISPRS J. Photogramm. Remote Sens..

[34] Fassnacht F.E., Hartig F., Latifi H., Berger C., Hernández J., Corvalán P., Koch B. (2014). Importance of sample size, data type and prediction method for remote sensing-based estimations of aboveground forest biomass. Remote Sens. Environ..

[35] Kattenborn T., Maack J., Faßnacht F., Enßle F., Ermert J., Koch B. (2015). Mapping forest biomass from space—Fusion of hyperspectral EO1-hyperion data and Tandem-X and WorldView-2 canopy height models. Int. J. Appl. Earth Observ. Geoinf..

[36] Latifi H., Nothdurft A., Koch B. (2010). Non-parametric prediction and mapping of standing timber volume and biomass in a temperate forest: Application of multiple optical/LiDAR-derived predictors. Forestry.

[37] Tian X., Li Z., Su Z., Chen E., Tol C.V.D., Li X., Guo Y., Li L., Ling F. (2014). Estimating montane forest above-ground biomass in the upper reaches of the Heihe River Basin using Landsat-TM data. Int. J. Remote Sens..

[38] Chen B., Wu Z., Wang J., Dong J., Guan L., Chen J., Yang K., Xie G. (2015). Spatio-temporal prediction of leaf area index of rubber plantation using HJ-1A/1B CCD images and recurrent neural network. ISPRS J. Photogramm. Remote Sens..

[39] Bright B.C., Hicke J.A., Hudak A.T. (2012). Estimating aboveground carbon stocks of a forest affected by mountain pine beetle in Idaho using lidar and multispectral imagery. Remote Sens. Environ..

[40] Chen C., Zhu J. (1989). The Manual of Forest Biomass in the Northeast of China.

[41] Lu D. (2006). The Potential and Challenge of Remote Sensing–Based Biomass Estimation. Int. J. Remote Sens..

[42] Breiman L. (2001). Random Forests. Mach. Learn..

[43] Pal M. (2005). Random forest classifier for remote sensing classification. Int. J. Remote Sens..

[44] Latifi H., Koch B. (2012). Evaluation of most similar neighbour and random forest methods for imputing forest inventory variables using data from target and auxiliary stands. Int. J. Remote Sens..

[45] Gu H., Dai L., Wu G., Xu D., Wang S. (2006). Estimation of forest volumes by integrating Landsat TM imagery and forest inventory data. Sci. China.

[46] Chirici G., Barbati A., Corona P., Marchetti M., Travaglini D., Maselli F., Bertini R. (2008). Non-parametric and parametric methods using satellite images for estimating growing stock volume in alpine and Mediterranean forest ecosystems. Remote Sens. Environ..

[47] Mcroberts R.E., Tomppo E.O. (2007). Remote sensing support for national forest inventories. Remote Sens. Environ..

[48] Monnet J.M., Chanussot J., Berger F. (2011). Support Vector Regression for the Estimation of Forest Stand Parameters Using Airborne Laser Scanning. IEEE Geosci. Remote Sens. Lett..

[49] Verrelst J., Muñoz J., Alonso L., Delegido J., Rivera J.P., Camps-Valls G., Moreno J. (2012). Machine learning regression algorithms for biophysical parameter retrieval: Opportunities for Sentinel-2 and -3. Remote Sens. Environ..

[50] Tuia D., Verrelst J., Alonso L., Perez-Cruz F. (2011). Multioutput Support Vector Regression for Remote Sensing Biophysical Parameter Estimation. IEEE Geosci. Remote Sens. Lett..

[51] Camps-Valls G., Bruzzone L., Rojo-Alvarez J.L., Melgani F. (2006). Robust support vector regression for biophysical variable estimation from remotely sensed images. IEEE Geosci. Remote Sens. Lett..

[52] Panda S.S., Ames D.P., Panigrahi S. (2010). Application of vegetation indices for agricultural crop yield prediction using neural network techniques. Remote Sens..

[53] Zheng S., Cao C., Dang Y., Xiang H., Zhao J., Zhang Y., Wang X., Guo H. (2013). Retrieval of forest growing stock volume by two different methods using Landsat TM images. Int. J. Remote Sens..

[54] Chang K.T., Liang L.S., Yiu F.G., Wang R.Y. Estimation of carbon sequestration by using vegetation indices. Proceedings of the 2012 IEEE International Geoscience and Remote Sensing Symposium (IGARSS).

[55] Johnson R.W. (2001). An Introduction to the Bootstrap. Macromol. Crystallogr..

[56] R Development Core Team (2013). R: A Language and Environment for Statistical Computing.

[57] Wilson F.C., Scott A. (2008). Applied Calculus.

[58] Hungerford T.W., Shaw D.J. (2008). Contemporary Precalculus: A Graphing Approach.

[59] Deng S., Katoh M., Guan Q., Yin N., Li M. (2014). Estimating Forest Aboveground Biomass by Combining ALOS PALSAR and WorldView-2 Data: A Case Study at Purple Mountain National Park, Nanjing, China. Remote Sens..

[60] Basuki T.M., Skidmore A., Hussin Y., Van Duren I. (2013). Estimating tropical forest biomass more accurately by integrating ALOS PALSAR and Landsat-7 ETM+ data. Int. J. Remote Sens..

[61] Laurin G.V., Chen Q., Lindsell J.A., Coomes D.A., Frate F.D., Guerriero L., Pirotti F., Valentini R. (2014). Above ground biomass estimation in an African tropical forest with lidar and hyperspectral data. ISPRS J. Photogramm. Remote Sens..

